# Supplementary material for: Optimal Anisotropic Guided Filtering in retinal fundus imaging: A dual approach to enhancement and segmentation
Source: PLoS One. 2025 Jul 31;20(7):e0329533. doi: 10.1371/journal.pone.0329533 (PMC12312952; doi:10.1371/journal.pone.0329533)
Supplement: Attachment — Submitted filename: Response to Reviewers.docx [file pone.0329533.s002.docx]

The assertions of all comments given by two reviewers are incorporated in the revised article and highlighted with colours.

Assertion for the Reviewer 1 comments are highlighted with green colour.

Assertion for the Reviewer 2 comments are highlighted with aqua blue colour.

In some cases, where the same comment from one or more reviewers then we used discrete colours.

# Reviewer 1

The article introduces an Optimal Anisotropic Guided Filtering (OAGF) framework designed for retinal fundus image enhancement and segmentation. The study is well-targeted to address the challenges of non-invasive retinal vascular imaging, making it particularly relevant for applications in ophthalmology and early diagnosis of systemic diseases. The methodology is sound, integrating illumination correction, optimized top-hat transform, and homomorphic filtering, which contributes to robust image segmentation and enhancement.

The paper is technically rigorous, with a comprehensive analysis of existing techniques and quantitative performance evaluation using DRIVE and STARE datasets. The results convincingly demonstrate superior performance compared to other segmentation methods. However, several aspects could be improved. The lack of a real clinical validation limits the study’s applicability to practical medical settings. Additionally, while the mathematical formulations are detailed, certain sections are too dense, making them less accessible to readers outside the field of image processing. Lastly, the computational complexity of the proposed approach is not sufficiently discussed, which may affect real-time applicability in clinical diagnostics.

The authors should reflect the following arising issues to improve the quality of manuscript:

**Comment 1**: Expand Clinical Validation and Comparison with Manual Expert Segmentation.

The study primarily evaluates OAGF against existing computational methods but lacks validation against manually segmented clinical datasets or expert ophthalmologist assessments. Including this comparison would improve the credibility of the proposed method and its suitability for real-world medical applications.

**Assertion 1:** We thank the reviewer for this insightful suggestion. We fully agree that validating our proposed OAGF framework against expert ophthalmologist segmentations is critical for demonstrating clinical reliability and translational potential.

In response, we have expanded the manuscript to include a comparative analysis between the OAGF-based segmentations and manually annotated ground truths provided in the DRIVE and STARE datasets. Both datasets include vessel annotations made by trained clinical experts, which we now use as reference standards for our evaluation.

Specifically, we performed quantitative comparisons using standard metrics such as Dice Coefficient (DC), Intersection over Union (IoU), Precision (PR), Recall (RE), and F1 Score (F1S). As presented in Table 2 and visualized in Figure 4 of the manuscript, our method consistently demonstrates a high degree of alignment with manual expert segmentations, achieving:

- Dice Coefficients of 0.860 (DRIVE) and 0.854 (STARE)
- Precision values of 0.845 (DRIVE) and 0.834 (STARE)
- Recall values of 0.811 (DRIVE) and 0.801 (STARE)

These results indicate that OAGF not only outperforms existing algorithmic approaches but also aligns closely with expert annotations, thereby reinforcing its potential for clinical adoption.

Furthermore, we have added a dedicated paragraph in the *Results Discussion* section to emphasize this alignment and clarify that the ground truths used for benchmarking stem from expert ophthalmologists, serving as a proxy for clinical validation in the absence of new annotations.

**Comment 2**: Clarify Computational Complexity and Feasibility for Real-Time Applications.

The proposed framework involves multiple processing stages, including top-hat transform, guided filtering, and homomorphic filtering, which may increase computational cost. A discussion on runtime performance, memory requirements, and potential hardware acceleration (e.g., GPU optimization) should be added to assess its feasibility in clinical practice.

**Assertion 2:** We appreciate the reviewer’s valuable comment regarding computational efficiency and the practicality of deploying our method in real-world clinical workflows.

To address this, we have included a detailed discussion on runtime performance, memory requirements, and hardware acceleration possibilities in the revised manuscript under the section *4.1 Experimental Setup.*

Key clarifications included in the revised manuscript are:

- *Runtime Performance*: The complete OAGF pipeline including illumination correction, anisotropic guided filtering, top-hat transform, and homomorphic filtering takes on a workstation with an Intel Core i9-12900K CPU, 32 GB RAM, and an NVIDIA RTX 3090 GPU. The segmentation and enhancement stages are executed sequentially but optimized using matrix-based operations in MATLAB.
- *Memory Usage*: Peak memory usage during processing remains under 2 GB, making the approach compatible with most modern GPU-equipped systems used in clinical imaging environments.
- *Hardware Acceleration*: The OAGF algorithm benefits significantly from GPU parallelization, particularly in stages involving convolution (e.g., homomorphic filtering in the frequency domain) and structural patch computation. We explicitly utilized MATLAB's GPU acceleration capabilities for Fourier transforms and matrix multiplications.
- *Scalability*: The algorithm maintains consistent performance across image resolutions commonly encountered in fundus imaging (e.g., 565×584 for DRIVE, 700×605 for STARE), confirming its scalability.

**Comment 3**: Improve Readability of Mathematical Derivations and Methodology.

While the article provides a mathematically rigorous formulation of OAGF, some key equations and algorithmic steps are dense and challenging to interpret. Including more intuitive explanations, flowcharts, and illustrative diagrams would make the methodology more accessible to interdisciplinary researchers.

**Assertion 3:** We thank the reviewer for this helpful comment. We recognize the importance of ensuring clarity in the mathematical presentation, particularly for readers from diverse disciplinary backgrounds.

To address this, we have ensured that each key mathematical derivation in the manuscript is immediately followed by explanations of the terms and variables used. This includes definitions for gradient operators, diffusion coefficients, structural patch elements, and regularization parameters such as $t$, $dx$ and $\epsilon$. These clarifications are embedded directly after equations (1) through (5), making the methodology more digestible.

Similarly, all equation we made. We expanded and clarified the block diagram to include the three processing stages (illumination correction, segmentation, and enhancement) with labeled inputs, outputs, and key transformation steps. This visual overview now provides a clearer roadmap of the methodology. In Section 3, we have restructured the explanation to include bullet points and short descriptors that walk the reader through the three-stage pipeline. This improves navigation and makes it easier to follow the logic and data flow.

We hope these measures have improved the accessibility and readability of the technical content, making it more approachable for readers with clinical, engineering, or data science backgrounds.

**Comment 4**: Address Potential Limitations and Biases in the Dataset

The study relies on DRIVE and STARE datasets, which are standard but relatively small and homogeneous. Discussing potential biases, dataset variability, and the effect of image quality variations (e.g., different fundus camera settings, patient demographics) would strengthen the paper’s generalizability.

**Assertion 4:** We thank the reviewer for highlighting this important point. We acknowledge that the DRIVE and STARE datasets, while widely used and annotated by experts, have inherent limitations in size and diversity, which may affect the generalizability of our findings.

To address this, we have added a dedicated paragraph to the *Conclusion* section (Last paragraph of chapter 5 in the revised manuscript) outlining the potential biases and constraints of using these datasets

# Reviewer 2

The paper presents a promising approach to retinal image enhancement and segmentation using OAGF. However, improvements in readability, justification, experimental validation, and comparative analysis with deep learning methods are necessary to strengthen the contribution. By addressing these issues, the manuscript can significantly improve in clarity, rigor, and impact.

**Comment 1**: The manuscript contains several long and complex sentences that make comprehension difficult. Simplifying technical explanations and improving readability would enhance accessibility. Break down dense paragraphs into shorter, more digestible sections.

**Assertion 1:** We appreciate the reviewer’s feedback regarding the clarity and readability of the manuscript. In response, we have undertaken a thorough language revision to improve sentence structure and overall accessibility.

We modified dense paragraphs, particularly in the Introduction, Methodology (Sections 2 and 3), and Experimental Results sections into smaller, clearly segmented parts, each focusing on a single idea.

**Comment 2**: In abstract, the manuscript does not clearly specify which publicly available databases were used. Providing names (e.g., DRIVE, STARE, or CHASE-DB1) will help contextualize the results. Specify the databases used and include detailed performance metric comparisons.

**Assertion 2:** We appreciate the reviewer’s suggestion to explicitly name the datasets used in the abstract. To improve clarity and context, we have updated the abstract in revised manuscript to include the names of the two publicly available databases DRIVE and STAR which were used to evaluate the proposed OAGF framework.

**Comment 3**: Incorporate a concise summary of the key results or findings in the abstract to provide readers with an overview of the study's outcomes. This approach offers a more comprehensive understanding of the research and encourages readers to explore the detailed results within the full paper.

**Assertion 3:** We thank the reviewer for this valuable suggestion. To enhance the impact and clarity of the abstract, we have added a concise summary of the key experimental results, highlighting the superior performance of the proposed OAGF framework in comparison with existing methods.

We have briefly summarized the performance of our method in comparison to existing techniques in terms of standard metrics such as Dice Coefficient, Precision, Recall, and F1 Score. These updates make the contributions and validation more transparent at a glance.

**Comment 4**: The introduction is dense and could be structured more clearly to gradually introduce the problem and background before diving into specific enhancement and segmentation methods. Clearly define the problem statement before diving into technical discussions.

**Assertion 4:** We thank the reviewer for the insightful observation. In response, we have restructured the Introduction section to improve its clarity and logical flow. The revised version of the manuscript now follows a more reader-friendly progression.

In revised manuscript we have incorporated the following:

“Traditional and deep learning-based enhancement and segmentation techniques frequently fight to optimize noise removal and maintain vessel structure integrity. Diffusion methods are prone to blur tiny details and learning methods need huge amounts of diverse data and tend to generalize less well under poor imaging conditions. Hence, there is an acute need for an approach to enhance the quality of an image and segment fine vascular structures well under poor conditions.

To solve this, we introduce an Optimal Anisotropic Guided Filtering (OAGF) framework. In this approach, enhancement and segmentation are to be performed in one pass by utilizing structure adaptivity, direction smoothing and contrast-preserving filtering. The OAGF framework is intended to prevent spatial information loss and maintain microstructure of blood vessels”.

Additionally, we have Simplified several long sentences for better readability, Clarified how enhancement and segmentation challenges motivate the proposed unified framework.

**Comment 5**: In section (Introduction), it’s better to clarify the whole structure of the paper, which can be easier for the reader to understand what scenarios have been investigated or examined and what’s the main contribution this paper makes. For example, this paper is organized as follows: section 2 presents what and section 3 presents what… It’s suggested to give a brief overview of the entire paper in the introduction. It is better to cite any relevant survey article in the introduction section to give an overview of the topic. e.g. Khan et al. "A review of retinal blood vessels extraction techniques: challenges, taxonomy, and future trends." Pattern Analysis and Applications 22 (2019): 767-802.

**Assertion 5:** We thank the reviewer for this helpful suggestion. In response, we have made two key additions to the *Introduction* section of the revised manuscript.

A. *Paper Structure Overview*: We now include a paragraph at the end of the Introduction summarizing the organization of the manuscript. This guides the reader through the progression of topics, from methodology to experimental validation.
B. *Relevant Survey Citation*: We have cited the comprehensive review by Khan et al. (2019) (As reference [28]), which provides a valuable taxonomy and summary of challenges in retinal blood vessel extraction. This citation helps position our work in the broader context of ongoing research.

**Comment 6**: The novelty of OAGF compared to existing anisotropic guided filtering methods needs better articulation. The paper should emphasize why OAGF is superior in more detail beyond computational efficiency and adaptability. Provide a more detailed discussion on how OAGF improves upon existing methods mathematically and practically.

**Assertion 6:** We thank the reviewer for this valuable comment. We agree that emphasizing the novelty of the proposed OAGF method is essential.

To clarify, the manuscript already provides a detailed explanation of how OAGF advances beyond existing anisotropic guided filtering techniques, both mathematically and in practical applications. However, based on the reviewer’s feedback, we have now highlighted key portions of this explanation to make the contributions more explicit and easier to locate.

Additionally, Section 4 presents a comparative analysis using quantitative metrics (e.g., Dice Coefficient, IoU, Precision) that substantiate OAGF’s superior performance over recent methods, validating its practical advantages in preserving vessel integrity under challenging illumination conditions.

We included the following in the revised manuscript

“OAGF introduces a modified optimization strategy (Equation 5) where the coefficients $a_{k}$ and $b_{k}$ are dynamically derived using a gradient-weighted approach, enabling region-aware and directionally adaptive smoothing. This improves structural preservation in fundus images compared to conventional filters.”

We hope that these clarifications, now made more prominent in the text, effectively communicate the novel contributions of OAGF. We thank the reviewer again for prompting this improvement in the manuscript’s presentation.

**Comment 7**: The equations provided (e.g., Equations 1-3) lack sufficient explanation regarding their derivation and how they improve upon existing techniques. Introduce more intuitive explanations for key equations.

**Assertion 7:** We thank the reviewer for this important observation. We agree that intuitive explanations are essential to make the mathematical formulations more accessible to a broad audience.

In response, we have revised the explanation of equations in the revised manuscript.

After Equation (1) we included “ $DCM(\cdot)$ corresponds to the Diffused Conductance Model that controls how much diffusion (smoothing) occurs at each pixel. In high-gradient areas (edges), the conductance is low to preserve detail, while in flat regions, higher diffusion smooths out noise. This enables edge-preserving filtering, which is essential in medical images where vessel boundaries must be maintained.”

After Equation (2) we included “Rather than employing one gradient alone, we examine intensity differences in eight directions: north, south, east, west, and diagonals. Patch-level representation of gradients makes the model sensitive to local edge orientations so more precise detection is facilitated in thin and curved vessels' structures. It imitates clinicians' visual evaluation of vascular patterns from several perspectives.”

**Comment 8**: The explanation of the weight function in equation (6) could be expanded with more intuitive descriptions of its impact on filtering.

**Assertion 8:** We thank the reviewer for this helpful suggestion. To enhance clarity, we have expanded the explanation following Equation (6) in revised manuscript to include an intuitive description of the weight function used in the filtering process.

The revised text highlights how the two exponential terms in the weight function operate:

- One governs intensity similarity, promoting edge preservation.
- The other governs spatial proximity, encouraging locality-aware smoothing.

This clearer interpretation will help readers understand how the filter balances contrast enhancement and noise suppression in diagnostically relevant regions.

**Comment 9**: The study lacks an explicit discussion on the computational complexity of the OAGF filter compared to other methods. Hybrid approaches integrating OAGF with CNN-based feature extraction should be investigated.

**Assertion 9:** We thank the reviewer for this important and forward-looking comment. We agree that both computational feasibility and potential hybridization with deep learning frameworks are critical aspects for practical deployment.

To address this, we have included a detailed discussion on runtime performance, memory requirements, and hardware acceleration possibilities in the revised manuscript under the section *4.1 Experimental Setup.*

Key clarifications included in the revised manuscript are:

- *Runtime Performance*: The complete OAGF pipeline including illumination correction, anisotropic guided filtering, top-hat transform, and homomorphic filtering takes on a workstation with an Intel Core i9-12900K CPU, 32 GB RAM, and an NVIDIA RTX 3090 GPU. The segmentation and enhancement stages are executed sequentially but optimized using matrix-based operations in MATLAB.
- *Memory Usage*: Peak memory usage during processing remains under 2 GB, making the approach compatible with most modern GPU-equipped systems used in clinical imaging environments.
- *Hardware Acceleration*: The OAGF algorithm benefits significantly from GPU parallelization, particularly in stages involving convolution (e.g., homomorphic filtering in the frequency domain) and structural patch computation. We explicitly utilized MATLAB's GPU acceleration capabilities for Fourier transforms and matrix multiplications.
- *Scalability*: The algorithm maintains consistent performance across image resolutions commonly encountered in fundus imaging (e.g., 565×584 for DRIVE, 700×605 for STARE), confirming its scalability.

*Potential for Hybrid CNN-OAGF Frameworks:*

We appreciate the reviewer’s suggestion regarding hybrid approaches. In future work, we plan to explore the integration of OAGF as a pre-processing or attention-enhancing module in CNN architectures. Specifically:

- OAGF can enhance vessel visibility and suppress irrelevant noise prior to feature extraction, improving CNN convergence and interpretability.
- Alternatively, OAGF could serve as a structural guidance module to reinforce feature maps in U-Net-style backbones.

We have briefly outlined this opportunity in the *Conclusion* as a future direction with strong potential for advancing clinical-grade retinal analysis.

**Comment 10**: While the DRIVE and STARE datasets are commonly used in retinal imaging research, the robustness of the method should be tested on additional datasets like CHASE_DB1 or HRF for further validation.

**Assertion 10:** We thank the reviewer for highlighting the importance of further validating the proposed OAGF framework on additional datasets such as CHASE_DB1 and HRF, which offer greater variability in terms of image resolution, camera systems, and patient demographics.

We acknowledge this as a valuable opportunity to demonstrate the generalizability and robustness of our method. While the current study focuses on DRIVE and STARE, which provide high-quality, expert-annotated ground truths suitable for initial benchmarking, we recognize that these datasets are limited in scope.

To address this, we have added a statement in the *Conclusion* section emphasizing the need for future evaluation on more diverse datasets such as CHASE_DB1 (with pediatric images and challenging illumination) and HRF (with high-resolution fundus scans). These datasets will help test OAGF’s adaptability across varied clinical conditions and imaging scenarios.

**Comment 11**: It would be beneficial to conduct an ablation study to assess the individual contributions of OAGF, Top-Hat Transform, and Homomorphic Filtering. Demonstrating performance variations when each component is removed or replaced would provide deeper insight into the necessity of each step. Highlighting specific cases where the proposed method performs significantly better (or worse) than contemporary methods can provide further insights.

**Assertion 11:** We thank the reviewer for this insightful recommendation. We fully agree that an ablation study would enhance the interpretability and analytical depth of the proposed OAGF framework by quantifying the individual contributions of each component OAGF, optimized Top-Hat transform, and Homomorphic Filtering.

In response, we have included a new ablation analysis in the section “**4.3.1 Segmentation results analysis”** with side heading “**Ablation Study and Component-Level Analysis**”.

**Comment 12**: The result section overwhelmingly highlights the superiority of OAGF without adequately discussing its limitations. For example, it is noted that Segmentation-3 and Segmentation-4 struggle with finer vessel details, but no insight is provided into why this occurs or how it might be addressed. Introduce a more balanced critique by acknowledging the challenges of OAGF, such as potential issues in handling extreme low-contrast images or cases with significant noise.

**Assertion 12:** We thank the reviewer for this thoughtful and constructive suggestion. We agree that acknowledging the limitations of the proposed OAGF framework is important for presenting a balanced evaluation and guiding future work. In response, we have incorporated limitations in conclusion section.

**Comment 13**: While Table 2 presents performance metrics, no statistical significance tests (e.g., t-tests, ANOVA) are conducted to validate whether OAGF's improvements are statistically meaningful. Include statistical tests to confirm whether the observed performance gains are significant or within the margin of error.

**Assertion 13:** We thank the reviewer for this thoughtful suggestion. While we did not explicitly conduct statistical significance tests such as *t-tests* or *ANOVA*, we would like to clarify that our study includes a comprehensive quantitative analysis of the proposed OAGF method compared to existing approaches.

Specifically, we evaluated performance using multiple well-established metrics, Dice Coefficient, Intersection over Union (IoU), Precision, Recall, and F1 Score across two benchmark datasets (DRIVE and STARE). These metrics were computed on a per-image basis, and results were averaged and reported in Table 2 to ensure consistency and reproducibility.

This detailed quantitative assessment was intended to offer a clear view of performance trends across diverse test cases. However, we acknowledge that incorporating formal statistical testing would provide additional validation. We appreciate the reviewer’s insight and will consider integrating statistical significance analysis in future extensions of this work.

**Comment 14**: The discussion asserts that OAGF “outperforms all existing methods,” but does not explain the algorithmic reasons for its success in depth. While it is clear that OAGF achieves higher accuracy, a more technical explanation of why it excels over deep learning-based methods (e.g., IUNet) is missing. Elaborate on the key architectural or mathematical advantages of OAGF that lead to its improved performance.

**Assertion 14:** We thank the reviewer for this thoughtful comment. We would like to clarify that a technical explanation for the performance advantages of OAGF is already provided in the Conclusion section of the manuscript. In that section, we describe how the directionally adaptive diffusion, structural patch analysis, and illumination correction mechanisms allow OAGF to outperform learning-based models, particularly in challenging scenarios such as uneven illumination or low-contrast vessel regions. These design elements contribute to superior edge preservation, noise suppression, and structural continuity in the segmented output, without relying on large-scale annotated datasets or complex training pipelines.

That said, in light of the reviewer’s feedback, we have slightly refined the wording in the conclusion to ensure these architectural advantages are more clearly emphasized.

We sincerely appreciate the reviewer’s suggestion, which helped improve the clarity and completeness of our final remarks.

**Comment 15**: The enhancement analysis mentions that OAGF “strikes the perfect balance” between detail preservation and contrast improvement. However, there is minimal discussion on how it achieves this balance compared to methods like NSCT or CLA. Provide a deeper technical explanation of how OAGF optimizes contrast and detail enhancement while minimizing artifacts.

**Assertion 15:** We thank the reviewer for raising this important point. While our results already demonstrate that OAGF achieves strong performance in both global contrast enhancement and local structural preservation, we agree that a more technical explanation of the underlying mechanism will better support the claim.

In response, in the revised manuscript, we have expanded the enhancement analysis in the Results Discussion section to explain how OAGF achieves this balance compared to NSCT, CLA, and other techniques.

The included paragraph is “The proposed framework of OAGF strikes a good balance between enhancing global contrast and preserving fine detail by utilizing a blend of gradient-aware diffusion and luminance-aware processing. In contrast to the uniform enhancement of the contrast by CLA at the expense of local detail degradation, OAGF performs edge-aware smoothing in the Y channel of the YCbCr color space. This provides selective enhancement of low-intensity vessel regions and avoids excessive enhancement of already bright regions. Additionally, structural patch-based anisotropic diffusion in OAGF dynamically adjusts the smoothing locally in the direction of gradient directionality, keeping high-contrast boundaries of the vessels and denoising the background regions effectively. Compared to the more conventional approach of fixing the directions in the sub-bands in NSCT, the use of localized, directionally aware filtering by OAGF prevents artifacts of the type of halos and ringing, hence the method is able to foreground-enhance both global and microvascular detail without inducing perceptual distortions.”

**Comment 16**: Figure 3 and Figure 5 provide valuable insights, but their descriptions lack detailed interpretations. For instance, it is mentioned that morphological filtering results in a “sparse, fragmented extraction,” but a more detailed explanation of how this affects clinical usability would strengthen the analysis. Improve the figure descriptions by explicitly linking the observed segmentation/enhancement quality to medical relevance and practical applicability.

Include some relevant papers in the comparison of results. For example https://journals.plos.org/plosone/article?id=10.1371/journal.pone.0158996

**Assertion 16:** We thank the reviewer for this insightful comment. We have highlighted the clinical relevance in the revised manuscript. The above said article, we referenced this study while outlining classical retinal vessel segmentation techniques, highlighting its role in combining Gabor filtering with morphological operations. The citation was used to support the historical context and motivate the need for more adaptive and structurally-aware approaches such as OAGF.

While we have not included it in the comparative results section, we believe the citation as reference number [38] in revised manuscript in the section 3.2 serves its purpose in positioning our contribution within the broader literature.
