# Supplementary material for: Optimal Anisotropic Guided Filtering in retinal fundus imaging: A dual approach to enhancement and segmentation
Source: PLoS One. 2025 Jul 31;20(7):e0329533. doi: 10.1371/journal.pone.0329533 (PMC12312952; doi:10.1371/journal.pone.0329533)
Supplement: Attachment — Submitted filename: Response_to_Reviewers_auresp_2.docx [file pone.0329533.s003.docx]

**Response to Reviewers Letter**

**Manuscript Title:** *Optimal Anisotropic Guided Filtering in Retinal Fundus Imaging: A Dual Approach to Enhancement and Segmentation*

Dear Editor and Reviewers,

We would like to thank you for your careful and insightful comments. We have revised the manuscript thoroughly in response to all reviewer suggestions. Below, we provide a point-by-point response to each comment. All changes have been incorporated into the revised manuscript and highlighted where appropriate.

In compliance with data transparency and reproducibility standards, we have uploaded the minimal dataset necessary to replicate our experimental findings to a stable public repository “Figshare” at the following link.

**URL: https://figshare.com/s/90317d14f5c06e69015b**

**DoI: 10.6084/m9.figshare.29277227**

We thank the editor and reviewers once again for their thoughtful feedback, which has significantly improved the quality of our work.

Sincerely,
**Dr. Venkataramana Guntreddi**
(on behalf of all authors)
email: gvramana@kiu.ac.ug

**Point by Point Response**

**Reviewer 1**

**Comment 1:** I have rerevised the modified manuscript. In my opinion, the authors have improved the modified manuscript in a sufficient manner to study meets standards and quality of scholarly papers. I am the opinion, that the paper has scientific rigor and well completes the state-of-the-art in the area retinal image segmentation.

**Assertion 1:** We sincerely thank the reviewer for their positive evaluation and encouraging feedback. We are grateful for your acknowledgment of the improvements made in the revised manuscript. Your constructive suggestions throughout the review process were instrumental in enhancing the clarity and quality of our work.

**Reviewer 3**

**Comment 1:** Authors have made all possible changes and reply all comments and no more further queries are required.

**Assertion 1:** We sincerely thank the reviewer for their thorough evaluation and confirmation that all comments have been satisfactorily addressed. We truly appreciate your constructive feedback and support throughout the revision process, which has helped us significantly improve the quality and clarity of the manuscript.

**Reviewer 2**

**Assertion for the Reviewer 2 comments are highlighted with aqua blue colour in the revised manuscript.**

The authors have addressed the majority of the previous' comments; however, several points still require more precise and comprehensive clarification.

**Comment 1:** The Introduction section introduces the OAGF framework and outlines the rest of the paper’s structure, which aligns with reviewer suggestions. However, the following shortcomings are still present:

Long, unbroken paragraphs make comprehension difficult. Break large paragraphs into shorter ones for better readability.

**Assertion 1:** We thank the reviewer for this helpful observation. In response, we have carefully restructured the manuscript.

**Comment 2:** The novelty of OAGF over other guided filtering techniques is not clearly highlighted (e.g., how does it differ from anisotropic diffusion or other edge-preserving methods?). Add a subsection or paragraph clearly stating how OAGF differs from existing filters, both mathematically and practically.

**Assertion 2:** We thank the reviewer for raising this important point. In response, we have explicitly clarified the novelty of the proposed **Optimal Anisotropic Guided Filter (OAGF)** and how it differs from conventional filtering techniques, including anisotropic diffusion (AD), bilateral filtering (BF), guided filtering (GF), and anisotropic guided filtering (AGF), both mathematically and practically. A dedicated subsection has now been added to the manuscript as “2.1	Distinctive features of OAGF vs. existing edge-preserving filters”, see Section 2, that outlines these differences and highlights the unique contributions of OAGF.

**Comment 3:** Consider summarizing traditional vs. deep learning segmentation shortcomings in a table or bullet list for clarity.

**Assertion 3:** We thank the reviewer suggestion to enhance clarity, we present a concise comparison of the limitations of traditional and deep learning-based retinal image segmentation approaches in the revised manuscript (in end of section 1 as table 1).

**Comment 4:** The OAGF is introduced as an “optimal” enhancement to AGF, but the specific mathematical or architectural innovations (e.g., how coefficients are “optimized” or how it differs in design principles from AGF) are not clearly articulated in contrast with prior work. Add a bullet-point or tabular comparison of mathematical innovations. Explicitly state what is new about your “optimal” weighting compared to AGF.

**Assertion 4:** We thank the reviewer for this insightful suggestion. In the revised manuscript, we have now added a new subsection titled “2.1 Distinctive features of OAGF vs. existing edge-preserving filters”.

**Comment 5:** Table 1, while useful, is qualitative and lacks metrics or empirical backing.

**Assertion 5:** We thank the reviewer for this observation. The intention of Table 1 is to provide a concise qualitative summary of edge-preserving filters (BF, GF, AD, AGF) based on their characteristics reported in prior literature [Refs. 29–32], not as a standalone experimental comparison. This summary helps readers grasp the relative strengths and limitations of existing methods before introducing our proposed OAGF.

To address the concern about empirical support, we clarify that extensive quantitative analysis of the proposed OAGF is already included in Tables 3, 5 and 6 (in revised manuscript). These tables benchmark OAGF against state-of-the-art segmentation and enhancement methods on the DRIVE and STARE datasets using standard metrics such as PSNR, SSIM, BRISQUE, DC, F1 Score, etc.

**Comment 6:** What happens in very low-contrast regions or high-noise inputs? Is the method robust to anatomical variability across datasets?

**Assertion 6:** We appreciate the reviewer’s thoughtful concern regarding robustness. The proposed OAGF framework is specifically designed to handle low-contrast and high-noise retinal images through its gradient-aware, anisotropic smoothing mechanism, which ensures fine vessel structures are preserved even under poor illumination. This is evident in our experimental results (Fig. 2, Segmentation-5 and Segmentation-6) where TS-5 and TS-6 represent challenging cases with uneven lighting and low clarity. The OAGF continues to preserve major and minor vessel continuity under such conditions, as confirmed by high segmentation scores (DC = 0.854, F1S = 0.817 on STARE).

Additionally, the use of both the DRIVE and STARE datasets which differ in terms of acquisition conditions, image resolutions, and retinal characteristics helps validate the method's cross-dataset robustness. Quantitative results in Tables 3 and 6 show consistently strong performance across these datasets.

**Comment 7:** Briefly explain how homomorphic filtering enhances vessel contrast by targeting frequency differences typical of vascular edges versus background, or include a visual before/after comparison.

**Assertion 7:** We thank the reviewer for this insightful suggestion. In response, we have added a brief explanatory paragraph to Section 3.2 (Stage 2) that clarifies the mechanism by which homomorphic filtering enhances vascular contrast. Specifically, homomorphic filtering operates in the log-frequency domain, suppressing low-frequency components associated with uneven illumination (background) and enhancing high-frequency components that correspond to vessel edges and fine anatomical structures. This selective frequency manipulation allows for better vessel enhancement prior to segmentation. We have also referenced this explanation alongside Equation (13) in the manuscript.

**Comment 8:** Include discussion on where the method might struggle (e.g., extremely low-contrast images, overlapping vessels, bright lesions mimicking vessel intensity).

**Assertion 8:** We thank the reviewer for this critical and constructive feedback. We have now expanded the discussion section of the manuscript to include a paragraph highlighting potential limitations of the proposed OAGF method (at end of section 4). Specifically, we acknowledge that while OAGF performs robustly in standard fundus images, it may face challenges in extremely low-contrast scenarios, where gradient-based edge cues are inherently weak. Additionally, the method may produce false positives in cases where bright lesions or exudates closely resemble vascular intensities, and it may struggle to distinguish overlapping or closely spaced vessels without topological cues. We have also identified these as areas for future enhancement, possibly through hybrid integration with deep learning-based discriminators or topological priors.

**Comment 9:** While numerous metrics (BRISQUE, NIQE, PIQE, IMMSE, PSNR, SSIM) are reported across multiple test samples, no statistical significance tests (e.g., t-test, Wilcoxon signed-rank test) are applied to confirm that the differences between OAGF and baseline methods are not due to random variation. Apply paired t-tests, Wilcoxon signed-rank, or ANOVA to key metrics (e.g., DC, IoU, F1) across test images to establish statistical superiority.

**Assertion 9:** We thank the reviewer for this valuable suggestion. In response, we have conducted paired t-tests and Wilcoxon signed-rank tests on a random subset of test images from the DRIVE and STARE datasets. These tests were applied to three key segmentation metrics: F1 Score (F1S), Dice Coefficient (DC), and Intersection over Union (IoU), comparing the proposed OAGF method against four baseline techniques: MDTF, IUNet, RVTHS, and MFFS. (We have used “ttest” and “signrank” commands in MATLAB to perform t-tests and Wilcoxon signed-rank tests ).

The results, summarized in the table below, demonstrate that OAGF achieves statistically significant improvements in all three metrics. In particular, the t-test p-values were all below 0.0001, and the Wilcoxon signed-rank test consistently returned p = 0.00195, indicating consistent directional superiority of OAGF across all evaluated test cases.

|  | F1S | | DC | | IoU | |
| --- | --- | --- | --- | --- | --- | --- |
|  | t-test p | Wilcoxon p | t-test p | Wilcoxon p | t-test p | Wilcoxon p |
| OAGF vs MDTF | 0.00001 | 0.00195 | 0.00002 | 0.00195 | 0.00002 | 0.00195 |
| OAGF vs IUNet | 0.00002 | 0.00195 | 0.00009 | 0.00195 | 0.00009 | 0.00195 |
| OAGF vs RVTHS | 0.00001 | 0.00195 | 0.00001 | 0.00195 | 0.00001 | 0.00195 |
| OAGF vs MFFS | 0.00001 | 0.00195 | 0.00001 | 0.00195 | 0.00001 | 0.00195 |

These findings confirm that the performance improvements achieved by OAGF are not due to random variation and are statistically robust. The results have been included in the revised manuscript under Section 4.3.1.

**Comment 10:** Figures 4(i) and 4(ii) present only bar values without error margins. Add standard deviation bars or box plots to convey performance variability across test cases. Fig. 5 also lack of error maps.

**Assertion 10:** We appreciate the reviewer’s attention to statistical completeness. In response, we have updated Figures 4(i) and 4(ii) to include standard deviation error bars for each metric across all test samples in the DRIVE and STARE datasets. This provides a clearer view of the consistency and variability of the proposed method compared to other approaches.

|  |  |  |
| --- | --- | --- |
| (i) TS1 | (ii) APC | (iii) TS1-APC residual map |
|  |  |  |
| (iv) CLA | (v) TS1-CLA residual map | (vi) NSCT |
|  |  |  |
| (vii) TS1-NSCT residual map | (viii) DWT | (ix) TS1-DWT residual map |
|  |  |  |
| (x) CT | (xi) TS1-CT residual map | (xii) Proposed OAGF |
|  |  |  |
|  | (xiii) TS1-OAGF residual map |  |

Figure 5(A): Residual Maps of enhanced image and original image

We appreciate the reviewer’s suggestion regarding error map inclusion. Error maps (residual maps) of the source image and the existing and proposed methods are shown in Figure 5(A). However, we believe that residual or error maps are not essential in this context to demonstrate the effectiveness of the proposed enhancement method. The visual improvements in contrast, structural continuity, and vessel clarity are already well-illustrated through qualitative comparison in Fig. 7 (in the revised manuscript) and are further supported by extensive quantitative metrics (BRISQUE, NIQE, PIQE, PSNR, SSIM) in Tables 6 and 7 (in the revised manuscript).

Since homomorphic filtering and OAGF are designed to preserve perceptual structure rather than minimize pixel-wise reconstruction errors, residual maps (e.g., |Enhanced – Original|) may not effectively reflect clinical enhancement quality. Therefore, we chose not to include them to maintain clarity and relevance in the manuscript’s visual assessments.

We hope this clarification addresses the concern.

**Comment 11:** Although BRISQUE and NIQE scores are mentioned, the paper doesn’t specify what ranges indicate well vs. poor quality. Include interpretive guidance—e.g., "BRISQUE < 30 indicates good perceived quality.

**Assertion 11:** Thank you for this helpful suggestion. We have updated the manuscript to include interpretive guidance for key quality metrics such as BRISQUE, NIQE, and PIQE. Specifically, we now state that BRISQUE scores below 30 typically indicate good perceived quality, and scores below 20 are considered high-quality. For NIQE, lower scores (typically < 5) reflect natural image quality, and PIQE values below 50 are generally acceptable. These ranges are now clarified in the revised manuscript in Section 4.3.2 (just above the Table 6).

**Comment 12:** While the TS-4 and TS-6 limitations are noted, no visual examples or deeper failure mode analysis is shown (e.g., where thin vessels are lost, or false positives occur). Show side-by-side visual comparisons of failure cases and briefly hypothesize the cause (e.g., low contrast, noise).

**Assertion 12:** We thank the reviewer for this insightful comment. To address this, we have now included a visual comparison of a failure case (test sample 30 from the dataset) in the revised manuscript (section 4.3.1), using three views: the original fundus image, the ground truth vessel map, and the segmentation result from the proposed OAGF method.

As shown in Figure 5, a localized zoom-in is used to highlight a region in the upper temporal area of the retina where thin vessels are lost or partially disconnected in the OAGF result. This degradation is likely caused by a combination of:

- Low vessel-to-background contrast in the source image
- High-frequency noise components that obscure weak edge gradients
- Lack of structural reinforcement in regions with minimal illumination

While OAGF performs well on prominent vessels, it may under-segment microvasculature under poor visibility. The inclusion of multi-scale vessel priors or attention-based refinement modules will be explored in future work to address these limitations.

**Comment 13:** While several filter parameters (e.g., t,dx,ϵ) influence OAGF performance, there is no analysis of how sensitive the performance is to these hyperparameters. Include a brief sensitivity analysis or at least a statement acknowledging the need for parameter tuning in deployment scenarios.

**Assertion 13:** We thank the reviewer for pointing out this important aspect. While a full parameter sweep was beyond the scope of the current work, we acknowledge the influence of the key hyperparameters, namely the iteration stopping time 𝑡, directional regularization parameter 𝑑𝑥, and smoothness coefficient 𝜖 on the performance of OAGF. To address this, we have added a brief discussion as “Hyperparameters ($t,dx,\epsilon$) sensitivity analysis” in Section 4.3.1 of the revised manuscript.

**Comment 14:** The large tables (Table 4) are rich in data but lack mean ± standard deviation summaries or any interpretation of variance. Summarize each metric across samples with mean ± SD, and highlight statistically significant improvements in bold or with asterisks.

**Assertion 14:** We thank the reviewer for this excellent suggestion. In response, we have updated Table 4 (Table 6 in updated manuscript) to include mean ± standard deviation (SD) for each image quality metric across all test samples. This provides a clearer view of the consistency and variance of each enhancement technique. Statistically significant improvements achieved by the proposed OAGF method are highlighted using boldface.
