# Supplementary material for: Optimal Anisotropic Guided Filtering in retinal fundus imaging: A dual approach to enhancement and segmentation
Source: PLoS One. 2025 Jul 31;20(7):e0329533. doi: 10.1371/journal.pone.0329533 (PMC12312952; doi:10.1371/journal.pone.0329533)
Supplement: Attachment — Submitted filename: Response_to_Reviewers_auresp_3.docx [file pone.0329533.s004.docx]

**Response to Reviewers Letter**

**Manuscript Title:** *Optimal Anisotropic Guided Filtering in Retinal Fundus Imaging: A Dual Approach to Enhancement and Segmentation*

Dear Editor and Reviewers,

We would like to thank you for your careful and insightful comments. We have revised the manuscript thoroughly in response to all reviewer suggestions. Below, we provide a point-by-point response to each comment. All changes have been incorporated into the revised manuscript and highlighted where appropriate.

We thank the editor and reviewers once again for their thoughtful feedback, which has significantly improved the quality of our work.

Sincerely,
**Dr. Venkataramana Guntreddi**
(on behalf of all authors)
email: [gvramana@kiu.ac.ug](mailto:gvramana@kiu.ac.ug)

**Point by Point Response**

**Reviewer 1**

**Comment 1:** I am the opinion that the revised manuscript has undergone substantial revisions. Based on the author’s reactions and the highlighted modifications it seems to be the revised manuscript has acceptable quality and scientific rigor. Therefore, I am the opinion that can be accepted in the present form.

**Assertion 1:** We sincerely thank the reviewer for the positive and encouraging feedback. We truly appreciate your acknowledgment of the substantial revisions and your recognition of the scientific rigor and quality of our revised manuscript. Your constructive comments throughout the review process have been invaluable in refining and strengthening our work. We are grateful for your recommendation and are pleased that the manuscript now meets the standards for acceptance.

**Reviewer 2**

**Comment 1:** While the section “2.1.2 Structural Awareness…” elaborates on regional adaptivity and structural preservation, a direct, bullet-point/tabular comparison highlighting “what’s new in OAGF vs. AGF” in practical deployment terms would enhance clarity further.

**Assertion 1:** We thank the reviewer for this insightful suggestion. To enhance clarity and better communicate the contributions of our **Optimal Anisotropic Guided Filter (OAGF)** over the existing **Anisotropic Guided Filter (AGF)**, we have now included a new comparison table **(Table 3 in the revised manuscript)** that provides a **deployment-oriented, side-by-side summary** of key improvements. This table clearly delineates how OAGF enhances practical utility in retinal image segmentation and enhancement scenarios.

**Comment 2:** Although it is mentioned in the pipeline (Lines 43–44), the specific rationale for how homomorphic filtering enhances contrast via frequency separation is not clearly explained. A short paragraph or figure showing its effect on vessel contrast is still missing.

**Assertion 2:** Thank you for the valuable comment. A concise **paragraph** to directly address the reviewer's suggestion and highlight the role of homomorphic filtering with added clarity is present **after Equation (13)** and **before Equation (14)** in **Section 3.2, Stage 2**.

“Homomorphic filtering in the frequency domain enhances the visibility of vessels by dissociating the reflectance and illumination parts of the image. Vessel structures normally have high-frequency attributes, while background illumination and non-uniform lighting have low-frequency elements. By using a high-pass filtering in the logarithmic domain (as indicated by equation (13)), the technique inhibits low-frequency effects of lighting and enhances high-frequency reflectance information, including vascular structure edges. All this gives rise to a greater vessel contrast, facilitating more precise segmentation. The selective filtering in the frequency domain is responsible for sustaining the continuity of fine vessels even in poor lighting conditions.”

**Comment 3:** The limitations of prior filters are noted, but no discussion is present yet on where OAGF might fail (e.g., overlapping vessels, low-contrast lesions). No visual failure cases are included.

**Assertion 3:** We thank the reviewer for this important observation. In response, we have a dedicated sub section **“Failure Case Analysis”** in section 4.3.1, to explicitly highlight and illustrate the potential failure scenarios of the proposed OAGF framework. **Fig. 5** demonstrates a visual failure case involving low-contrast micro vessels.

**Comment 4:** Equations and detailed formulas are now included (e.g., Eq. 24–29). However, no interpretive scale (e.g., “BRISQUE < 30 = high quality”) or guidance for what counts as a good score is provided.

**Assertion 4:** We thank the reviewer for this important observation. In response, we have now added a paragraph (**in section 4.3.2, above Table 7**) summarizing what constitutes a “good” score for each metric to improve interpretability.

**“**More specifically, BRISQUE values below 30 would be considered good perceptual quality, while values below 20 are regarded as high quality with minimal distortions. NIQE values below 5 appear to depict good naturalness of an image with values below 4 being high visual fidelity. PIQE is a block-based measure of perceptual distortion such that lower scores signify better scores; values below 50 appear to be acceptable while values below 30 is indicative of enhanced image clearness. IMMSE is an error-based measure such that values below 0.1 appear to signify highly accurate restorative work with minimal deviation in ideal estimates. PSNR, in general used in image compression as well as enhancement works, is regarded as being acceptable at values above 30 dB with excellent quality at values above 50 dB. Lastly, SSIM values range between 0 and 1 such that scores above 0.95 appear to be regarded as excellent in structural similarity as well as feature conservation”

**Comment 5:** While parameters like t, dx, and ϵ are referenced in Eq. (9), there is still no discussion of sensitivity or robustness to these parameters.

**Assertion 5:** We thank the reviewer for raising this important point. We would like to clarify that a detailed sensitivity analysis of the parameters t, dx, and ϵ is presented in the manuscript under Section 4.3.1, and the corresponding results are illustrated in **Figure 6(i–iii).**

**Comment 6:** While the section 4.3.2 compares OAGF against a set of conventional image processing methods (CLA, DWT, NSCT, etc.), it does not include or reference recent unsupervised or hybrid segmentation techniques, which are highly relevant for enhancing retinal vessel visibility. <https://doi.org/10.1155/2020/8365783> Add a runtime analysis or mention the average processing time per image compared to other methods. Consider citing and briefly discussing the following paper in Section 4.3.2 (suggest after line 710 or in limitations at line 775): Khan, K.B., Khaliq, A.A. and Shahid, M., 2017. “A Novel Fast GLM Approach for Retinal Vascular Segmentation and Denoising,” Journal of Information Science and Engineering, 33(6), 1611–1627.

**Assertion 6:** We thank the reviewer for pointing out this relevant work. In response, we have updated the Introduction section **(reference [29] in the revised manuscript)** to cite the recommended study [DOI: 10.1155/2020/8365783], which presents a **hybrid model combining region-based enhancement with unsupervised segmentation**, showing promise in preserving vascular structures without requiring ground-truth labels.

We have incorporated **(reference [60] in the revised manuscript)** a brief discussion and citation of the paper by **Khan et al. (2017)**. The study proposes a **fast generalized linear model (GLM)-based approach** for simultaneous vessel segmentation and denoising, which offers computational efficiency and robustness in noisy retinal images. While the GLM method focuses on statistical modeling and pixel-level classification, the proposed OAGF approach is based on gradient-driven structural adaptation and frequency-domain enhancement.

Regarding runtime, while we have provided general insights into the computational feasibility of the proposed OAGF framework in Section 4.1 highlighting its GPU compatibility and low memory footprint we acknowledge that a detailed runtime benchmarking across methods was not included in the current manuscript. We agree that such analysis would offer important practical insight into comparative efficiency. We therefore plan to incorporate a comprehensive runtime evaluation in our future work, including per-image processing time comparisons across classical, transform-based, and deep learning approaches. This will help further assess the real-time suitability and scalability of OAGF in clinical settings and large-scale deployment scenarios.

**Comment 7:** Include standard statistical tests (e.g., paired t-tests or Wilcoxon signed-rank) across metric distributions to verify that observed improvements by OAGF are statistically significant and not due to sample variance.

**Assertion 7:** We thank the reviewer for this valuable suggestion. We would like to highlight that standard statistical validation has already been conducted and reported in the manuscript under Section 4.3.1, with sub heading of ***“Statistical significance tests”***. Specifically, we performed both paired t-tests and Wilcoxon signed-rank tests as **listed Table 5**.

**Comment 8:** Add one paragraph discussing how improved microvascular clarity (via OAGF) supports diagnosis of diabetic retinopathy, glaucoma, or hypertensive retinopathy.

**Assertion 8**: We appreciate the reviewer’s insightful suggestion. In response, we have added the following paragraph at **end of the results section (Section 4).**

“The improved fine retinal vasculature visualization facilitated by the presented OAGF framework is extremely valuable in early disease detection and in monitoring of systemic as well as ocular diseases. In diabetic retinopathy, early signs like microaneurysms, dropout of capillaries, and neovascularization happen at the microvascular scale and can be inadvertently overlooked in poorly enhanced images. In glaucoma, as well, assessment of the retinal nerve fiber layer as well as localized thinning of vessels near the optic disc is improved with increased vessel-edge contrast. In hypertensive retinopathy, fine details such as narrowing of arterioles, arteriovenous nicking, as well as hemorrhages, demand high-fidelity structural detail in order to aid in accurate grading. By maintaining vessel continuity as well as highlighting capillary-level structures, OAGF enhances diagnostic conspicuity as well as allows for improved clinical interpretation.”

**Comment 9:** The results are derived solely from DRIVE and STARE datasets. While they are standard, these datasets lack diversity in pathological variations and image acquisition conditions. Mention this limitation and recommend validation on CHASE_DB1, HRF, or ARIA, which include pathological cases and varied resolutions. Also, consider stating the adaptability of OAGF across different imaging conditions.

**Assertion 9:** We thank the reviewer for highlighting this important consideration. We admit as well that while widely available and with high-quality expert annotation, the DRIVE as well as STARE datasets suffer significantly in diversity of pathology, image resolution, as well as variability of acquisition conditions. That can reduce generalizability of experimental results. To counter this, we have now mentioned this limitation in text of this revised manuscript in the Conclusion section. We propose further as future research using diverse datasets like CHASE_DB1, HRF, as well as ARIA, with larger variability of pathological condition (e.g., diabetic retinopathy, hypertensive retinopathy) as well as realistic variability of image quality as well as image resolution. We would moreover particularly refer to mention as well that in principle transferable OAGF framework developed here is of gradient-driven, region-aware functional framework formulation, independent of dataset-specific priors as well as of supervised learning. This inherent structural independence is indicative of good generalizing potential of OAGF across imaging conditions, albeit such empirical verification as a future work is needful in this direction.
